# Clitoral size in term newborns in Kumasi, Ghana

**DOI:** 10.1186/s13633-017-0045-y

**Published:** 2017-06-06

**Authors:** Serwah Bonsu Asafo-Agyei, Emmanuel Ameyaw, Jean-Pierre Chanoine, Margaret Zacharin, Samuel Blay Nguah

**Affiliations:** 1 Department of Child Health, Komfo Anokye Teaching Hospital, Kumasi, Ghana; 2 Endocrinology and Diabetes Unit, British Columbia Children’s Hospital, University of British Columbia, Vancouver, BC Canada; 3 The Royal Children’s Hospital, Murdoch Children’s Research Institute and University of Melbourne, Melbourne, Australia

**Keywords:** Clitoral length, Clitoral width, Clitoral size, Ghana, Newborns, Clitoromegaly

## Abstract

**Background:**

Data on normative clitoral sizes in newborns is relatively sparse and racial/ethnic differences have also been reported. This study was performed to establish norms for clitoral size in term Ghanaian female newborns.

**Methods:**

This was a cross-sectional study of all apparently well full-term newborns of postnatal age < 48 h and birth weight between 2.5 and 4.0 kg delivered at Komfo Anokye Teaching Hospital between May and September, 2014. Anthropometric and genital parameters were documented for study subjects as well as parental socio-demographic indices.

**Results:**

In 612 newborn females studied, the mean (±SD) clitoral length (MCL) and the mean (±SD) clitoral width (MCW) were 4.13 ± 1.6 mm and 4.21 ± 1.1 mm, respectively. MCL was inversely related to birth weight (*r* = −0.62; ***p < 0.001***) while MCW was inversely related to both gestational age (*r* = −0.1; ***p = 0.02***) and birth weight (*r* = −0.54; ***p < 0.001***). Babies with a clitoris that was completely covered by the labia majora had relatively lower clitoral sizes (*p* < 0.001) than those who had a partially covered or prominent clitoris. Neither MCL nor MCW differed significantly by birth length or maternal tribe.

**Conclusions:**

Clitoral size varies with birth weight and gestational age. Babies with a completely covered clitoris are unlikely to warrant detailed clitoral measurements for clitoromegaly.

## Background

Early detection of genital anomalies in the female infant is crucial. Significant clitoromegaly at birth usually reflects virilization and suggests that the female foetus has been exposed to androgens during the intrauterine period [1]. Congenital adrenal hyperplasia (CAH) is the most common cause of virilization of a female foetus. Failure to identify and treat this disorder results in potentially fatal adrenal crisis, an avoidable outcome if the endocrine emergency is correctly diagnosed and treated [2]. Since the technique of analyzing 17-hydroxyprogesterone (17-OHP) in filter paper blood samples was developed by Pang et al. [3] in 1977, the utility of newborn screening for CAH has been amply demonstrated and several developed countries have established a newborn screening programme [4–7]. Unfortunately, newborn screening is still not available in most developing countries including Ghana. Thus, until routine newborn CAH screening is commenced, comprehensive clinical assessment including genital examination remains the simplest and most cost-effective option of circumventing the challenges imposed by financial and diagnostic restraints in resource limited settings.

Propitiously, females with classical CAH usually have ambiguous genitalia or clitoromegaly which may be readily detected by genital examination [2]. Hospital statistics at the study site indicate that about 9000 - 12,000 babies are delivered annually in the hospital, with about 16 - 18% of these being preterms. Newborn screening for congenital adrenal adrenal hyperplasia is not carried out in the hospital but between May 2014 and April 2015 we diagnosed 4 females with CAH in a cohort of 9255 neonates through systematic newborn examination (unpublished data). Although it has been reported that clinical examination may miss some cases of non-classical and even classical CAH [5, 8, 9], detailed genital examination still has distinct benefits over a cursory look at the genitalia and may even help prevent extremely virilized females from being labelled as “males”.

Conversely, the clitoris may seem prominent in some healthy newborns, leading to many unnecessary investigations [10]. Clitoral size measurements permit accurate clitoral assessment and avoid over- or misdiagnosis of abnormalities based on clinical impression alone, thus minimizing unnecessary cost and psychological trauma to the parents [11].

Normative clitoral anthropometric data for healthy newborns has been reported but is sparse and mainly derived from Caucasian and Asian infants. However, racial/ethnic differences in newborn clitoral sizes have been reported [12, 13] and existing data may therefore not be applicable to our population in Ghana, for which no published data exist.

Aim: To establish reference ranges for clitoral sizes in apparently healthy term newborns in Ghana.

## Methods

This cross-sectional study was conducted in Komfo Anokye Teaching Hospital (KATH) between May 2014 and September 2014. KATH is a tertiary care teaching hospital in Kumasi, the second largest city in Ghana. It serves as a referral centre for the Kumasi Metropolis and the whole northern half of Ghana.

A complete antenatal history was obtained from the mother and from the hospital records, including a history of ingestion of herbal medicine or prescribed medications. The gestational age was determined by using the last menstrual period and early ultrasound results and confirmed if necessary with Dubowitz/Ballard score [14]. Physical examination of both mother and newborn was done. All apparently well female term newborns with a gestational age of 40 ± 2 weeks and birth weight between 2.5 and 4.0 kg were considered for recruitment into the study. Exclusion criteria for newborns included major congenital anomalies/dysmorphism, apparent disorders of sexual development, breech presentation and twins of different gender. Maternal exclusion criteria were pregnancy history of hormonal drug intake, signs of virilization during pregnancy, pre-eclampsia and diabetes mellitus.

**Fig. 1:**
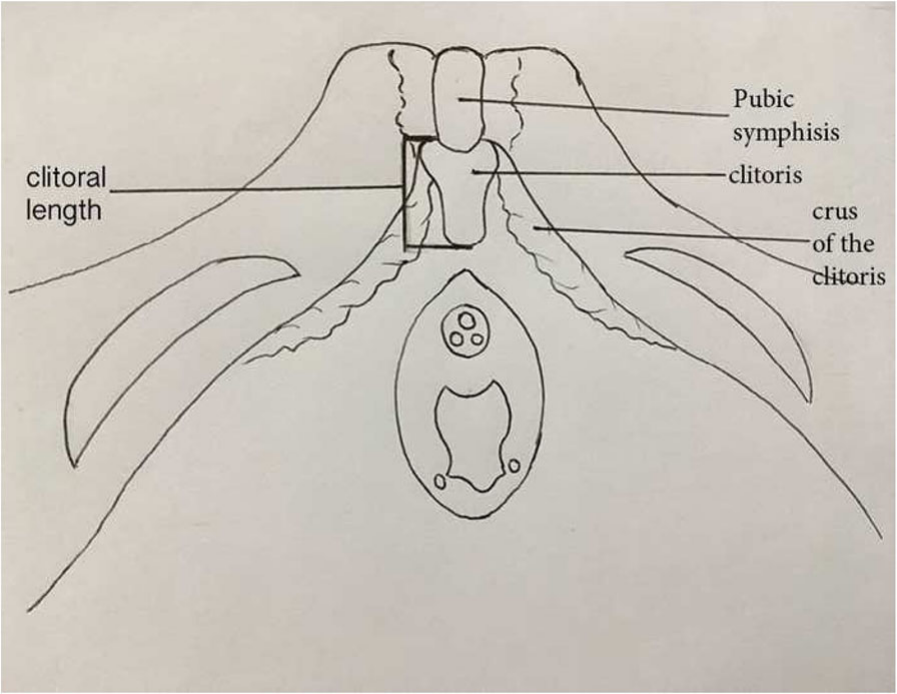
Schematic diagram of clitoris and surrounding structures showing the anatomic landmarks utilized for clitoral length measurement

Anthropometric measurements of newborns were taken within 48 h after delivery. A nurse attached to the paediatric endocrine unit was trained to assist the principal investigator with the genital and anthropometric measurements. Two research assistants were also trained to assist the two examiners with positioning of the newborns and with data entry. In a warm environment, the newborns were put in the supine position and the perineum was adequately exposed. It was noted on inspection whether the clitoris was visible or completely covered by the labia majora. The baby was then placed in a frog-like position and the position secured by an assistant. The labia majora were separated and the prepuce of the clitoris gently retracted. Clitoral size was measured as described by Verkauf et al. [15], with clitoral length measured as the distance from the crura insertion at the pubis symphysis to the tip of the glans and clitoral width measured in the greatest transverse diameter (Fig. 1). Both the clitoral length and width were measured twice using digital Vernier calipers (Resolution 0.01 mm, Accuracy +/−0.02 mm) and their respective means were recorded. The weight was measured to the nearest 10 g with a Salter scale (Model 180, Salter Brecknell, England) and the length was measured to the nearest centimetre with an infantometer (Seca 416 Mobile infantometer, Seca GmbH & Co. KG, Germany). All anthropometric measurements were done by a 2-member team, with one person taking the measurements while the other positioned the baby and helped enter the data onto the Case report form. Majority (72.1%) of the measurements were done by the principal investigator. Inter-observer variability for genital measurements was checked on a 2-weekly basis throughout the study on 5 randomly selected newborns and it remained insignificant. The standard deviations for inter-observer variation were 0.05 mm and 0.09 mm and 95% of paired measurements were within range of 0.09 mm and 0.03 mm for clitoral length and clitoral width respectively.

The data was analyzed using R statistical software version 3.1.2. Continuous variables were presented as means with standard deviation as well as median with their corresponding ranges. Single categorical variables were tabulated and expressed as percentages. The relationships between genital measurements and various categorical variables were determined using an analysis of variance, correcting for possible confounders and reporting the respective *p*-values as appropriate. Relationships between genital measurement and other continuous variables were determined using linear regression. These analyses were presented as their regression coefficient (r) with their 95% confidence intervals. For all analysis a two sided *p*-value of <0.05 was considered statistically significant. This study was approved by the Committee on Human Research, Publications and Ethics of KATH/Kwame Nkrumah University of Science and Technology, Kumasi. Written informed consent was received from the parents prior to enrollment.

## Results

**Fig. 2:**
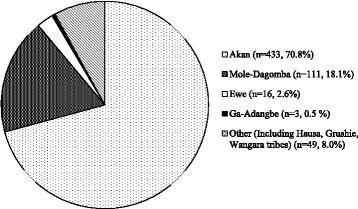
Distribution of maternal tribe in study subjects

A total of 612 female infants were studied including one set of identical twins. One mother/baby pair was excluded because of maternal virilization. A descriptive data of study subjects is shown in Table 1. The distribution of maternal tribe is shown in Fig. 2. Genital measurements from study subjects were used to construct clitoral size percentile charts (Table 2). The mean (±SD) clitoral length [MCL] was 4.13 ± 1.6 mm and the mean (±SD) clitoral width [MCW] was 4.21 ± 1.1 mm. There were 3/612 females (0.49%) with a clitoral length ≥ 1 cm and 31/612 female newborns (5.1%) with a clitoral width greater than 6 mm. Using the 97^th^ percentile, a newborn will be considered to have clitoromegaly if clitoral length is greater than 7.5 mm and/or clitoral width is greater than 6.2 mm.

**Table 1:** Descriptive characteristics of study subjects

	Mean	SD	Median	Minimum	Maximum
Gestational age *(weeks)*	40.0	1.1	40.0	38.0	42.0
^a^Post-natal age *(hours)*	10.1	7.8	8.0	1	47
Birth weight (*kgs*)	3.1	0.4	3.1	2.5	4.0
Length *(cm)*	48.1	2.1	48.2	37.6	53.6
Clitoral Length (*mm*)	4.1	1.6	3.7	1.1	10.8
Clitoral Width (*mm*)	4.2	1.1	4.3	1.3	8.8

^a^Age in hours at which anthropometric measurements were done

**Table 2:** Clitoral size percentiles

Percentile	Clitoral Length (mm)	Clitoral Width (mm)
3	1.6	2.2
10	2.3	2.8
50	3.9	4.3
90	6.3	5.6
97	7.5	6.2
99	8.6	6.8

No correlation was seen between genital size and maternal herbal intake or mode of delivery. Seventy-nine (12.9%) mothers took herbs at varying periods during their pregnancy. There was also no significant difference in genital measurements done in the first 24 h of postnatal life and those done from 24 h up to 48 h. After correcting for confounders, there was no significant difference in MCL or MCW by tribe. Table 3 shows the correlation between clitoral size and gestational age, birth weight and birth length. There was a strong negative correlation between clitoral size and birth weight.

**Table 3:** Association of clitoral size with gestational age, birth weight and birth length

	Gestational Age (*weeks*)		Birth Weight (*kgs*)		Birth Length (*cm*)	
	*r* (95% CI)	*p*-value	*r* (95% CI)	*p*-value	*r* (95% CI)	*p*-value
MCL *(mm)*	−0.06 (−0.17 to 0.06)	0.330	−0.62 (−0.96 to −0.29)	***<0.001***	−0.03 (−0.09 to 0.03)	0.390
MCW *(mm)*	−0.1 (−0.17 to −0.02)	***0.020***	−0.54 (−0.78 to −0.31)	***<0.001***	−0.04 (−0.08 to 0)	0.070

Linear regression utilized for analysis; r: Coefficient of linear regression

Significant *p*-values are in bold italics

Both clitoral length and width varied significantly with appearance of the clitoris (Table 4). A completely covered clitoris was significantly smaller than a partially covered one, which was in turn smaller than a prominent clitoris.

**Table 4:** Correlation between clitoral appearance and size

^a^Clitoris	Completely covered number =132 (21.6%)		Partially showing number =188 (30.7%)		Prominent number = 278 (45.4%)		*p*-value
	Mean (SD)	Median (range)	Mean (SD)	Median (range)	Mean (SD)	Median (range)	
CL (mm)	3.3 (1.2)	3.3 (1.1–8.3)	3.8 (1.4)	3.8 (1.4–10.8)	4.8 (1.6)	4.8 (1.2–10.6)	***<0.001***
CW (*mm*)	3.5 (1.0)	3.5 (1.3–6.2)	3.8 (0.9)	3.8 (1.8–6.1)	4.8 (1.1)	4.8 (2.0–8.82)	***<0.001***

Analysis of variance utilized for analysis; Significant *p*-values are in bold italics

^a^Fourteen missing data

## Discussion

This is the first description of the characteristics and size of the clitoris in Ghana and the largest cohort of newborn clitoral sizes in published literature. Traditionally, normal clitoral length is accepted as < 1.0 cm, although rare variations exist [16]. In our study, only 3/612 females (0.49%) had a clitoral length ≥ 1 cm. These patients were followed up for a year and their clitoral size did not increase with time, nor did they develop any clinical features suggestive of CAH. Studies in newborns from different parts of the world have reported MCL ranging from 3.1–7.7 mm [10, 13, 17–21]. Most published studies done in newborns did not elaborate on how the clitoral measurements were done. Amongst studies with similar inclusion criteria, our MCL of 4.13 ± 1.60 mm was comparable to the 4.0 ± 1.24 mm reported by Oberfield et al. [17] in United States and the 4.93 ± 1.61 mm reported by Kutlu et al. [10] in Turkey. Our MCL was also similar to that of other studies that involved preterms, including the 3.66 ± 0.13 mm reported by Riley and Rosenbloom [13] in black term and preterm newborns in United States. Mondal et al. [20] also reported a comparable albeit slightly lower MCL of 3.1 ± 1.54 mm in India and Jarrett et al. [19] reported a relatively higher MCL of 7.7 ± 1.37 mm in 244 term and preterm newborns in Nigeria.

A clitoral width > 6 mm has been said to suggest virilization [22], probably based on earlier studies by Riley and Rosenbloom (1980) [13] and Oberfield et al. (1989) [17]; who both reported an upper limit of 6 mm for clitoral width range in newborns. However, the range for clitoral width is not well established as fewer studies have reported clitoral width. In this study, as many as 31 / 612 newborns (5.1%) had a clitoral width greater than 6 mm. These patients were followed up and they remained healthy. Jarrett et al. [19] also reported a clitoral width range of 1–7 mm, and so regional differences in clitoral size may exist. More African data will be needed to clarify this. Our MCW of 4.21 ± 1.1 mm was similar to reports from Jarrett et al. [19] and Yokoya et al. [21] of 4.4 ± 0.89 mm and 4.4 ± 1.2 mm respectively. Oberfield et al. [17] reported a relatively lower mean clitoral width (MCW) of 3.32 ± 0.78 mm in North American term newborns.

Factors that may be responsible for the reported variation in clitoral sizes include differences in study population. Some studies included preterms [13, 19, 20] whereas this study recruited only term newborns. Clitoral size has also been reported to correlate with anthropometric parameters of the study population [10]. Furthermore, differences in tools and techniques of measurement as well as inter observer variability may play a role. This study utilized calipers in the measurement of clitoral length and width while other studies utilized tumorimeters or rulers [19, 23]. MCL have also been reported to vary with ethnicity/race, which may also partly account for the differences in reported values [12, 13].

Only MCW but not MCL in our study was negligibly associated, though inversely, with gestational age. The lack of significant association was not unexpected as all the newborns were full term. The analysis for correlation between maternal herbal intake and clitoral size was done to evaluate for possible hormonal effects of the ingested herbal medicines, since their constituents were largely unknown. There was no significant difference in genital measurements done in the first 24 h and those done afterwards. Oberfield et al. [17] also noted no difference in clitoral sizes when they compared measurements done before and after 24 h in order to evaluate for possible variation from swelling of the genital area due to birth trauma.

Birth weight was inversely associated with both MCL and MCW, implying newborns with a lower birth weight had larger clitoral sizes and vice versa. Similarly, Kutlu and Akbiyik [10] also found a negative correlation between MCL with birth weight. However, unlike this study they also reported a negative correlation between MCL and birth length. Oberfield et al. [17] found no correlation between clitoral size and birth weight or length.

Kutlu and Akbiyik [10] reported that the clitoral length was < 5 mm when it appeared to be covered by the labia majora and concluded that no extra clitoral measurement was clinically indicated in such cases. In our study, the highest recorded MCL and MCW in newborns with completely covered clitoris was 8.3 mm and 6.22 mm respectively. Overall, newborns whose clitoris was completely covered by the labia majora had the lowest mean clitoral size (*p* < 0.001) and indeed further clitoral measurements may not be indicated in such newborns; as suggested by Kutlu and Akbiyik [10].

One limitation of our study is that measurements were done by two examiners and could have introduced an error due to inter-observer variability. However, an extensive training was done and a subset of study subjects was examined by both 2 examiners to evaluate and control for variability, which remained insignificant throughout the study.

## Conclusions

Our study suggests that the mean clitoral size of Ghanaian newborns is similar to results reported by others but regional differences may exist in clitoral size range. Our data will be useful for the assessment of female neonates in Ghana by establishing the norm and thus help promote early recognition of deviation from the norm; such as genital abnormalities in female neonates.

## Abbreviations

CAH: Congenital Adrenal Hyperplasia
CI: Confidence Interval
KATH: Komfo Anokye Teaching Hospital
MPL: Mean Penile length
MPW: Mean Penile Width
r: Coefficient of linear regression
SPL: Stretched penile length
